# Structural connectome with high angular resolution diffusion imaging MRI: assessing the impact of diffusion weighting and sampling on graph-theoretic measures

**DOI:** 10.1007/s00234-018-2003-7

**Published:** 2018-03-08

**Authors:** Giuseppina Caiazzo, Michele Fratello, Federica Di Nardo, Francesca Trojsi, Gioacchino Tedeschi, Fabrizio Esposito

**Affiliations:** 1 MRI Research Center SUN-FISM – Neurological Institute for Diagnosis and Care “Hermitage Capodimonte”, 80131 Naples, Italy; 2 Department of Medical, Surgical, Neurological, Metabolic and Aging Sciences, University of Campania “Luigi Vanvitelli”, Naples, Italy; 3 Magnetic Resonance Imaging Research Center of the Second University of Naples-Italian Foundation for Multiple Sclerosis, Second University of Naples, Naples, Italy; 4 Department of Medicine, Surgery and Dentistry, Scuola Medica Salernitana, University of Salerno, Via S. Allende, 84081 Baronissi, Salerno Italy; 5 Department of Cognitive Neuroscience, Faculty of Psychology and Neuroscience, Maastricht University, 6201BC Maastricht, The Netherlands

**Keywords:** Diffusion MRI, Tractography, Networks, Connectivity, Gradient sampling schemes, Connectome

## Abstract

**Purpose:**

Advances in computational network analysis have enabled the characterization of topological properties of human brain networks (connectomics) from high angular resolution diffusion imaging (HARDI) MRI structural measurements. In this study, the effect of changing the diffusion weighting (*b* value) and sampling (number of gradient directions) was investigated in ten healthy volunteers, with specific focus on graph theoretical network metrics used to characterize the human connectome.

**Methods:**

Probabilistic tractography based on the Q-ball reconstruction of HARDI MRI measurements was performed and structural connections between all pairs of regions from the automated anatomical labeling (AAL) atlas were estimated, to compare two HARDI schemes: low *b* value (*b* = 1000) and low direction number (*n* = 32) (LBLD); high *b* value (*b* = 3000) and high number (*n* = 54) of directions (HBHD).

**Results:**

LBLD and HBHD data sets produced connectome images with highly overlapping hub structure. Overall, the HBHD scheme yielded significantly higher connection probabilities between cortical and subcortical sites and allowed detecting more connections. Small worldness and modularity were reduced in HBHD data. The clustering coefficient was significantly higher in HBHD data indicating a higher level of segregation in the resulting connectome for the HBHD scheme.

**Conclusion:**

Our results demonstrate that the HARDI scheme as an impact on structural connectome measures which is not automatically implied by the tractography outcome. As the number of gradient directions and *b* values applied may introduce a bias in the assessment of network properties, the choice of a given HARDI protocol must be carefully considered when comparing results across connectomic studies.

## Introduction

Brain connectivity and the connectome have unlocked new experimental and theoretical avenues in many areas of neuroscience. Recent advances in diffusion magnetic resonance imaging (dMRI) and functional magnetic resonance imaging (fMRI) techniques have made it possible to model the human brain as a complex network in vivo. Using graph theory, both a functional and a structural brain network can be described as a graph, i.e. a collection of nodes, each corresponding to a brain region, and connections or edges, each expressing a pathway between two nodes. On the basis of a suitable formal description, a number of graph-theoretic measures can be used to describe several properties of the network’s architecture [1].

Structural connectivity can be assessed in vivo in humans by the combination of dMRI-based white matter tractography and gray matter parcellation methods. Compared to clinical applications of dMRI, increasing the diffusion weighting and the number of diffusion gradient directions represent two simple options available to any clinician in order to increase the angular resolution of white matter tractography and therefore the robustness of graph-theoretic measures. Although the effect of *b* value on HARDI reconstruction for tractography has been studied before [2], here we compared for the first time the impact on the estimation of network properties in two HARDI MRI protocols, one with a low *b* value and a low number of direction (LBLD) and one with a high *b* value and a high number of directions (HBHD).

The most typical graph-theoretic metrics used in brain connectomics include the degree of clustering, the global efficiency, the modularity, and the small worldness [3]. Briefly, the degree of clustering and the strength of a given node measure the extent to which the node is connected to the rest of the network, while the centrality and the efficiency capture how many short paths between two parts of the network pass through the node. Modularity refers to the property of a network of being divided into multiple modules according to how nodes result densely interconnected with other nodes within the same module and sparsely interconnected with other nodes outside the module. A “small-world” network is characterized by a topology in which most of the nodes are not neighbors of each other but can be reached through a relatively small number of steps [4]. Some works have revealed that brain networks intrinsically possess small world properties, and some small world attributes have been proposed as clinical markers [5–7].

To enable the calculation of these metrics and fully characterize the brain structural networks in terms of graph-theoretic measures, the diffusion tensor imaging (DTI) [8, 9] represents the most common post-processing technique for dMRI data to calculate the initial connectivity matrix, which represents the input of all graph-theoretic measures. However, HARDI acquisition techniques in combination with the Q-ball imaging (QBI) [10] have been more recently explored in the context of graph-theoretic characterizations of brain structural networks [3].

In this study, we performed a whole-brain connectomic analysis using a probabilistic QBI tractography on a cortical parcellation of 90 regions obtained from the automated anatomical label (AAL) atlas and determined the effect of the dMRI acquisition scheme on global and local graph network measures.

## Methods

### Subjects

Ten healthy subjects (4 male and 6 female, age 52 ± 7.15 years) were scanned on a 3T GE Medical System scanner (Signa HDxt3T twin speed GE) equipped with an eight-channel parallel head coil. The research was conducted according to the principles expressed in the Declaration of Helsinki. Ethics approval was obtained from the Ethics Committee of the institute where the study has been performed, and informed consent was obtained from all participants.

### MRI acquisition

Three-dimensional T1-weighted sagittal images were acquired with GE sequence IR-FSPGR (TR = 6988 ms, TI = 1100 ms, TE = 3.9 ms, flipangle = 10,voxel size = 1 mm × 1 mm × 1.2 mm).

Whole-brain diffusion-weighted MRI was performed first using a spin echo echo-planar imaging (EPI) LBLD sequence (repetition time = 10,000 ms, echo time = 83.2 ms, field of view = 320 mm, isotropic resolution = 2.5 mm, *b* value = 1000 s/mm, 32 isotropically distributed gradients, frequency-encoding left-right (LR)) and then with a spin echo EPI HBHD sequence (repetition time = 16,000 ms, echo time = 104 ms, field of view = 320 mm, isotropic resolution = 2.5 mm, *b* value = 3000 s/mm, 54 isotropically distributed gradients frequency-encoding LR).

### Data preprocessing

Motion, eddy currents correction and brain tissue extraction (BET) of diffusion-weighted images were performed with FSL version 5.0.8 [11]. A Q-ball model was fitted at each voxel, generating generalized fractional anisotropy (GFA) maps with qboot, a command line tool of the FSL package. This command allows estimation of diffusion ODFs using the Q-ball constant solid angle (CSA) model [12]. After coregistration of dMRI images with T1-weighted images, the cortical gray matter parcellation was performed using the Automated Anatomical Labeling Atlas (AAL) [13, 14] which includes 90 cortical and subcortical regions. The obtained structures were then used as ROIs for fiber tracking. Probabilistic fiber tracking was performed in FSL according to Behrens and colleagues [15]. To estimate the connection probability, probabilistic tractography was applied by sampling 5000 streamline fibers per each voxel. For each sampled fiber, a sample direction was first drawn from the local direction distribution at the seed voxel, then a new sample direction from the local distribution was obtained at the next position, located 0.5 mm along the previous direction, etc. For each seed region, 5000 × n fibers were sampled, *n* being the number of voxels in the region. The number of fibers passing through a given region divided by 5000 × *n* is finally given as the connection probability from the seed region to the target region [16]. In the present study, each cortical region was selected as the seed region and its connection probability to each of the other 90 regions was calculated [17].

### Network construction and graph-theoretic measures

From the tractography results, each data set was transformed into a connectivity matrix, measuring connection probability from the seed region to the target region. Each individual network is thus represented by a symmetric 90 × 90 matrix, in which each row and column represents a node and each element represents an edge.

The raw individual networks are likely to contain spurious connections due to noise and algorithm errors; however, the graphs can be controlled for spurious connections using group-level non-parametric statistics [18]. In fact, connections between two specific nodes are more likely to be real and reliable if they are consistently detected across individuals. The non-parametric sign test was applied by taking each individual as a sample, with the null hypothesis being that there is no existing connection (i.e., connectivity weight = 0). The Bonferroni method was used to correct for multiple comparisons across all node pairs within the network. For each group data set (LBLD, HBHD), the node pair surviving a corrected *p* < 0.05 was deemed to have a connection. As a result, a binary matrix (1 for node pairs with a connection and 0 for node pairs without a connection) was generated for each group of WM networks. This binary mask was then applied to each individual subject network to remove the spurious connections [19]. For each type of WM networks, the network density, which is the fraction of present connections to possible connections, becomes the same across all subjects of the groups, improving the between-groups comparability of network measures. We calculated eight network measures as described in [20] with the Brain Connectivity Toolbox: Betweeness Centrality, Global efficiency, Local efficiency, Node strength, Node degree, Clustering coefficient (C), Characteristic Path-length (L), Modularity (M), and Small worldness. Each of these properties and their biological significance has been defined and discussed in detail elsewhere [1, 6]. Moreover, the mean betweenness centrality was calculated for each node in LBLD and HBHD groups, respectively. Then, regions on the topographic betweenness centrality map with values in the 80th percentile were defined as group hub regions [21, 22].

### Statistical analysis

The network-based statistic (NBS) tool [23] was used to measure the size of sequence effect using intensity of network connectivity values comprising pairs of regions after correction for multiple comparisons. For each nodal and global scale, the null hypothesis LBLD = HBHD was tested using a *t* test and was rejected with *p* < 0.05 for all network measures considered (false discovery rate (FDR) corrected).

## Results

**Fig. 1:**
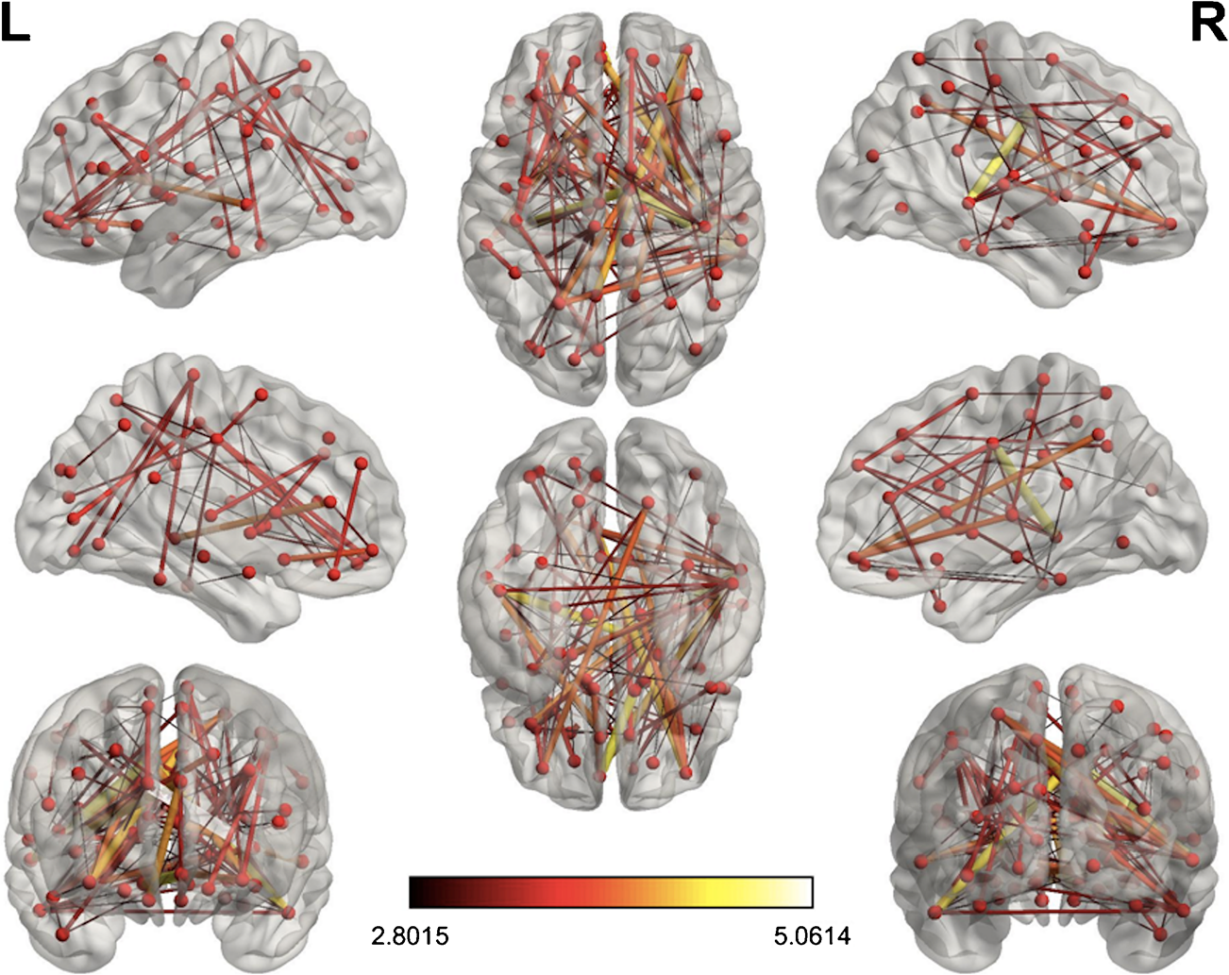
Representation of nodes and edges showing significantly higher connection probability (*t* score) in HBHD compared to LBLD data sets (*p* < 0.05, corrected for multiple comparisons)

NBS analysis revealed a significantly higher connection probability in HBHD compared to LBLD data sets in 88 pairs of node (*p* = 0.0002) (Fig. 1, Table 1).

**Table 1:** Connections showing significantly higher connection probability (*t* score) in HBHD compared to LBLD

Seed region	Target region	*T* score (9 df) of links
Frontal_Sup_L	Supp_Motor_Area_R., Rectus_R., Temporal_Mid_R.	2.88, 2.88, 3.81
Frontal_Sup_R	Frontal_Sup_Medial_R., Insula_L., Angular_R., Temporal_Mid_R.	2.83, 3.26, 3.28, 3.28
Frontal_Sup_Medial_L	Rectus_L., Rectus_R., Fusiform_R., Temporal_Mid_R.	3.54, 4.13, 3.3, 4.19
Frontal_Mid_L	Rectus_R., Temporal_Mid_R.	3.01, 2.96
Frontal_Mid_R	Insula_L., Angular_R., Temporal_Mid_R.	3.09, 2.91, 2.83
Frontal_Sup_Medial_R	Insula_L., Insula_R., Hippocampus_R., Fusiform_R., Caudate_L., Temporal_Mid_L., Temporal_Mid_R.	5.06, 2.86, 3.05, 3.11, 3.44, 2.81, 3.32
Frontal_Inf_Oper_L	Cingulum_Ant_R.	3.07
Frontal_Inf_Orb_L	Cingulum_Mid_R., Postcentral_R., Caudate_L.	4.03, 3.19, 3.22
Insula_L	Cingulum_Post_L., Cingulum_Post_R., Hippocampus_R.,Lingual_R., Fusiform_R., Postcentral_R.	2.84, 2.89, 3.88, 3.23, 2.97, 2.93
Rolandic_Oper_L	Hippocampus_R.	3.02
Cingulum_Mid_R	Occipital_Sup_L., Occipital_Mid_R., Parietal_Sup_L., Parietal_Sup_R., Putamen_R., Pallidum_R., Temporal_Mid_R., Temporal_Inf_L., Temporal_Inf_R.	3.53, 2.83, 3.96, 2.99, 3.52, 3.16, 4.67, 4.43, 3.17
Cingulum_Mid_L	Occipital_Mid_L., Parietal_Sup_L., Temporal_Mid_L., Temporal_Inf_L.	3.19, 3.02, 2.94, 3.32
Frontal_Sup_Orb_R	Fusiform_R., Temporal_Inf_R.	2.82, 2.9
Cingulum_Ant_L	Fusiform_R., Temporal_Mid_L., Temporal_Mid_R.	2.88, 3.99, 2.94
Calcarine_L	Postcentral_L.	3.68
Lingual_L	Postcentral_L., Paracentral_Lobule_L.	2.8, 3.34
Insula_R	Postcentral_R.	2.83
Cingulum_Post_R	Postcentral_R., Parietal_Inf_L., Temporal_Inf_R.	3.27, 2.89, 2.98
Supp_Motor_Area_R	Parietal_Sup_R.	2.98
Occipital_Inf_L	Paracentral_Lobule_L.	3.47
Fusiform_L	Paracentral_Lobule_L., Temporal_Sup_R.	3.53, 2.89
SupraMarginal_R	Paracentral_Lobule_L., Heschl_R.	2.81, 2.97
Frontal_Inf_Tri_L	Thalamus_R., Temporal_Mid_R.	2.87, 2.83
Thalamus_R	Temporal_Sup_R., Temporal_Mid_R.	3.66, 2.92
Cingulum_Ant_R	Temporal_Mid_L., Temporal_Pole_Mid_R., Temporal_Inf_L.	3.15, 3.5, 2.97
Caudate_R	Temporal_Mid_L., Temporal_Inf_L.	2.8, 2.94
Supp_Motor_Area_L	Temporal_Mid_R.	3.33
Cingulum_Post_L	Temporal_Mid_R., Temporal_Inf_R.	3.03, 3.72
Occipital_Sup_L	Temporal_Mid_R.	3.14
Occipital_Mid_L	Temporal_Mid_R.	3.14
Parietal_Sup_L	Temporal_Mid_R.	3.85
Putamen_L	Temporal_Pole_Mid_R., Temporal_Inf_R.	3, 3.27
Temporal_Pole_Sup_R	Temporal_Pole_Mid_R.	2.99
Amygdala_L	Temporal_Inf_L.	2.92
Rectus_R	Temporal_Inf_R.	3
Hippocampus_L	Temporal_Inf_R.	3.24
Parietal_Sup_L	Temporal_Inf_R.	3.15
Temporal_Inf_L	Temporal_Inf_R.	3.33

The global clustering coefficient was also found higher in HBHD compared to LBLD (*p* = 0.026).

Global M and small worldness were significantly lower in HBHD, compared to LBLD data sets (M: *p* = 0.001; small worldness: *p* = 0.042). There were no significant differences in global efficiency and L.

**Fig. 2:**
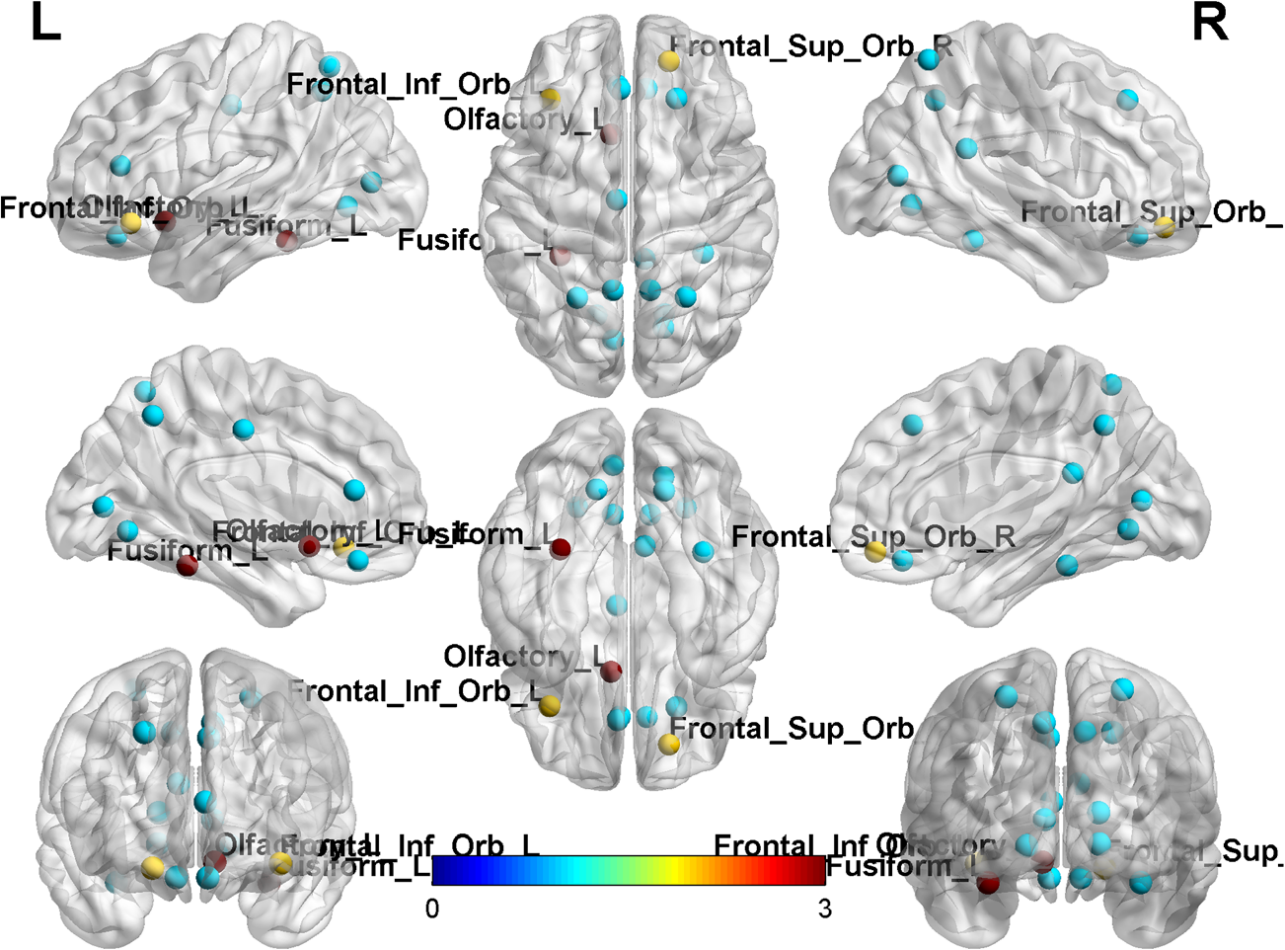
Hub regions with groups betweenness centrality scores in the 80th percentile displayed on brain MNI template surface. Blue nodes were hubs in common between the two-acquisition scheme; red nodes were hubs only for HBHD scheme and yellow only for LBLD scheme

The distribution of hubs across the whole connectome was similar between the two sequences. Hubs in common between LBLD and HBHD data sets are summarized in Table 2 (Fig. 2). Two regions corresponding to the left inferior frontal orbital gyrus and the right superior frontal orbital gyrus in the AAL were identified as hubs only in LBLD-derived matrices, and two regions corresponding to the left olfactory cortex and the left fusiform gyrus in the AAL were identified as hubs only in HBHD-derived matrices.

**Table 2:** List of hubs in common between the two data set (HBHD and LBLD) and in each group

Name of hub region	Acquisition scheme
Left superior frontal orbital	HBHD, LBLD
Left rectus	HBHD, LBLD
Left anterior cingulum	HBHD, LBLD
Left medial cingulum	HBHD, LBLD
Left calcarine	HBHD, LBLD
Right calcarine	HBHD, LBLD
Right lingual	HBHD, LBLD
Left lingual	HBHD, LBLD
Right fusiform	HBHD, LBLD
Left superior parietal	HBHD, LBLD
Right superior parietal	HBHD, LBLD
Left precuneus	HBHD, LBLD
Right precuneus	HBHD, LBLD
Right superior orbital frontal	LBLD
Left frontal inferior orbital	LBLD
Right posterior cingulum	HBHD, LBLD
Right superior frontal	HBHD, LBLD
Right rectus	HBHD, LBLD
Left fusiform	HBHD
Left olfactory cortex	HBHD

In terms of local measures, local efficiency was higher in HBHD in several regions (right temporal superior and medial gyri, bilateral medial cingulate cortex, right superior temporal, postcentral and anterior cingulate cortex, medial frontal gyrus, angular gyrus, and left inferior temporal gyrus) compared to LBLD data (*p* < 0.05, FDR corrected); local clustering coefficient was higher in HBHD in right temporal superior and medial gyrus, bilaterally medial cingulate cortex, right medial frontal and postcentral gyrus, anterior cingulate cortex, angular gyrus, left inferior temporal gyrus, and left and posterior cingulate cortex (*p* < 0.05, FDR corrected).

There were no significant differences in betweenness centrality, node strength, and node degree after FDR correction.

## Discussion

Graph theoretical measures can be used to characterize network topological organization in both healthy and diseased brains and to detect structural and functional abnormalities associated with different neurological and psychiatric disorders. These measures can be broadly classified into global measures, assessing the level of network integration, and local measures, assessing the level of network segregation.

Structural brain networks have been shown to be both highly segregated (due to the presence of relatively more selective pathways between specific regions compared to other) and highly integrated, i.e. forming several small-world networks or modules [24]. Both aspects could be independently altered in brain pathology with respect to normative values. In this work, we investigated the influence of the dMRI gradient scheme on the computational measures of structural brain networks obtained with QBI tractography at different numbers of directions.

We detected an overall increase in the values of connection probability across all pairs of anatomical regions when comparing the HBHD data set to the LBLD data set. This can be due to the fact that higher *b* values cause stronger diffusion weightings; however, due to the lower signal-to-noise, this may also create false positive connections because of the noise. To reduce the impact of false positives on subsequent network feature estimations, we only consider as actual connections (of varying strength) those connections that were consistently present across all subjects using a non-parametric procedure based on the sign test [19].

Another difference between HBHD and LBLD data sets that might have possibly contributed to the observed increase in the connection probabilities and the number of connections is the higher density of sampled directions of the HBHD scheme. In fact, given that the number of connections depends on how much the sampled direction agrees with the orientation of the fiber, the connection probability is expected to be lower for the LBLD scheme, [25, 26] . On the other hand, with a higher number of sampled directions in the HBHD scheme, it is more probable to measure higher signal responses as more sampled directions tend to correspond better to the actual orientation of the fibers.

Using the QBI, in line with [4], we found that the small world metrics were dependent on the directional resolution of the gradient scheme, with the values of small worldness in HBHD being significantly lower than LBLD data sets. This is in agreement with our expectation since a higher number of secondary tracts in addition to the main ones (that are in common between the LBLD and HBHD schemes) likely imply that brain modules are relatively more connected to each other via secondary connections than via direct connections (that pass through the network hubs).

LBLD also showed significantly higher values for M compared to HBHD, M being the degree to which a system can be broken into multiple subnetworks. This clearly indicates that LBLD data produce more segregated and less interconnected network modules. Conversely, lower M values are indicative of stronger connections among modules, thus gathering a more integrated distribution of subnetworks or modules.

The level of clustering expresses the level of local connectedness of a network, with high levels of clustering commonly interpreted as high levels of local organization of the network in modules [23]. Averaged over the entire network, the HBHD clustering coefficient was higher than that of LBLD, suggesting a higher degree to which relatively closer nodes share local connectivity within the module. In other words, HBHD data better highlighted the presence of densely interconnected groups of regions within each module.

Despite the higher number of connections detected for HBHD, the hub structure (i.e. the approximate number and location of nodes qualified as hubs) was highly similar between LBLD and HBHD data sets. Nonetheless, the lower M values obtained for HBHD suggest that the information about the overall transmission of neural impulses among modules is better preserved by the HBDB scheme (see, e.g., [1]).

Addressing the influence of the dMRI sequence parameters on graph-theoretic measures is therefore fundamental for the interpretation of various local and global structural brain network measures. In fact, differences in structural brain network measures could be mistakenly related to markers of neurological disease whereas these might be due to a different sensitivity of the particular acquisition scheme.

For DTI [4], the average node degree has been shown to increase with the number of gradient directions (6, 12, and 32), which may result from the fact that longer streamlines are tracked and, therefore, more densely connected networks are generated, at higher directional resolution. However, even if characteristic path length increases, the mean clustering coefficient drops when the number of gradients rises from 6 to 12 [4]. Indeed, DTI can only estimate a single fiber direction per imaging voxel, thereby the partial volume effect may potentially average two or more fiber populations with different local orientations across large parts of the imaging volume. This has stimulated the investigation of different acquisition protocols in combination with alternative water diffusion models also for graph-theoretic measures.

Similar trends were observed for the diffusion spectrum imaging (DSI) approach, which uses hundreds of diffusion sensitive gradients with different amplitudes to sample directions typically along a Cartesian grid. DSI has the potential of producing biologically more meaningful mapping of the human connectome; however, it requires long acquisition times (> 35 min) [26], thereby DSI can be not feasible for clinical applications. In contrast to DSI, QBI is a model-free reconstruction scheme which allows measuring the angular structure of the diffusion spectrum with less diffusion directions and therefore shorter acquisition times (< 20 min). The QBI model estimates the orientation distribution function (ODF) over a sphere sampled at many points, allowing the identification of more than one diffusion direction in each voxel and enabling a more accurate fiber tractography in regions with multiple populations of fibers with different orientations.

Tractography studies can reveal localized network abnormalities by investigating one or more specific white matter tracts [27], but in some diseases such as epilepsy [28], Alzheimer’s disease [24] and schizophrenia [4], the abnormality does not necessarily involve specifics white matter tracts, and the exact location of the impairment remains unknown. Nonetheless, it remains possible to assess the integrity of the entire brain network using graph theory. In a previous work [25], we investigated the impact of the QBI model on the tractography of major white matter tracts using different gradient acquisition schemes and showed that clinical studies aiming to investigate WM fiber integrity may benefit from application of a QBI model to dMRI data sets acquired at high *b* values and high numbers of diffusion direction (HBHD scheme) compared to low *b* values and low number of diffusion direction (LBLD scheme), especially when analyzing fiber tracts characterized by more than one dominant fiber direction. In previous work, we proposed an HARDI acquisition scheme with a *b* value and number of directions resulting in an acceptable scanning time (~ 15 min) for clinical applications [29] and demonstrated that this scheme can gather good performances at reasonable scan times for studying the (generalized) fractional anisotropy of WM regions [25]. Similarly, in the present work, we have shown important effects of the dMRI scheme also on global and local network features, which in some cases could be linked to the optimal sampling of fiber bundles with more than one dominant fiber direction. This was most likely the case for the anterior part of the fronto-occipital fasciculus (right superior frontal medial and right temporal medial nodes) and the superior longitudinal fasciculus (left insula and right hippocampus nodes), which where two regions where the local network properties exhibited significant differences between the two schemes. On the other hand, apart from the acquisition protocol, network metrics for the human connectome are also affected by several post-processing parameters, such as the orientation model, the brain parcellation, the tractography algorithm, and the weight threshold [3].

Several studies have revealed important topological properties for human brain WM networks [5, 17, 30], addressing the effects of brain abnormalities on specific network properties in terms of modified weight or threshold parameters [31–33]. For instance, in the “weighted graph” approach, a distance weight is associated with each edge linking two nodes; thus, by combining (e.g., averaging) connection matrices across multiple subjects, inconsistent or weak weights are confronted with ad hoc thresholds, and eventually removed, leading to a reduction of the connections. However, there is no standard threshold in the literature and its value is often a free choice value in the graph-theoretic analysis. Moreover, apart from the thresholds, the criterion used for cortical gray matter parcellation, the dMRI acquisition scheme, the diffusion data model, and the fiber tracking algorithm are all methodological variants that may have a substantial impact on the resulting network measures.

In conclusion, our results suggest that both local and global topological properties of human structural brain networks exhibit strong dependence on the choice of dMRI acquisition scheme, also when the Q-ball model is used for tractography. This adds up to previous similar evidence obtained on DTI schemes and may thus help the planning of future connectomic studies in specific pathological populations.

These results are potentially relevant if several pathologies are investigated, especially by comparing those mostly affecting subcortical structures (e.g., Parkinson’s disease and other movement disorders involving basal ganglia) versus those mostly affecting cortical structures (e. g., Alzheimer’s disease, frontotemporal dementia, amyotrophic lateral sclerosis, and psychiatric conditions), as supported by several recent connectomic analyses [34–39]. In particular, a connectomic approach could reinforce current knowledge of the interplay between the cerebral cortex, the basal ganglia, and the thalamus and its role in the pathophysiology of neurological disorders involving the cortico-basal and thalamo-cortical loops and their links to the cerebellum [37, 40]. Moreover, connectomic alterations of brain circuits may have a predictive role of clinical outcome in several neurological [38, 39, 41] and psychiatric [42] conditions.

Further investigation is needed to quantify the extent to which the reported results hold of a general population, as this study comprised a small data set of 10 subjects. Moreover, future studies are needed to compare different (non-QBI) dMRI models among them and possibly disentangle the effect of the *b* value and number of directions in the determination of graph-theoretic measures of structural brain networks.
